# Building the field of food systems research: commentary on a research funder’s role

**DOI:** 10.1186/s12961-021-00745-7

**Published:** 2021-07-16

**Authors:** Hayley Pelletier, Leah Bleecker, Victoria Sauveplane-Stirling, Erica Di Ruggiero, Daniel Sellen

**Affiliations:** University of Toronto, Toronto, ON M5T 3M7 Canada

**Keywords:** Field-building, Food systems, Research capacity-building, Health research

## Abstract

**Background:**

The Food, Environment, and Health (FEH) program of the International Development Research Centre (IDRC) aims to improve the health of low- and middle-income country populations by generating evidence, innovations, and policies that reduce the health and economic burdens of preventable chronic and infectious diseases. A predominant focus of the FEH program is research related to consumer food environments that promote or enable healthy and sustainable shifts in consumption. An evaluation of the FEH program, led by the University of Toronto, provided an opportunity to analyse the approach and role of a development funder in building the field of food systems research.

**Discussion:**

In this commentary, we provide an external evaluator’s perspective on the IDRC’s contributory role in building the field of food systems research, based on a secondary analysis of findings from a recent FEH program evaluation. We used the field-building framework outlined in Di Ruggiero et al. (Health Res Policy System, 2017) to highlight the strengths and challenges of the FEH’s approach to field-building and determined that the program aligns with six of the seven features of the framework. The FEH program has enhanced support and awareness for food systems research, provided organized funding and capacity-building opportunities, multilevel activity to support research and its use, and strong scientific leadership, and set significant standards and exemplars. However, we also found that not all sociopolitical environments have fully recognized or valued food systems research and its use for policy change.

**Conclusion:**

The FEH program’s field-building approach can be situated within the field-building framework, and it has been successful in laying the groundwork for building the field of food systems, particularly consumer food environments research. However, supportive external environments and further investments may be needed to achieve a critical mass of capacity, continue building communities of practice, and influence policy. The FEH program approach may serve as an exemplar and comparator for other research funding agencies looking to develop strategic research programming in the field of food systems research.

## Introduction

A research field consists of a group of individuals and organizations that work collectively to solve a shared problem or develop advancements in knowledge, policy or practice through research. Establishing a strong field of research is important for creating momentum to advance knowledge and to generate evidence that addresses problems in society [12].

The field-building framework described in Di Ruggiero et al. [4] outlines several key features needed to successfully build a field of research: support and awareness of the field, standards and exemplars, influential scientific leadership by a core cadre of researchers, and the presence of an organized funding stream. Additionally, it is important to have supportive environments to build research teams' capacity and multilevel activities to support the funding and use of research findings to advance the field and influence policy.

“Food systems” is a relatively new field of research that studies the interconnections and impacts of nutrition, health, agriculture, marketing, food packaging, and production, with a significant focus on promoting sustainability [10]. Relatedly, the International Development Research Centre (IDRC) [8] describes food systems research as including food policies, community-based food initiatives, food marketing, nutrition, and dietary changes [9]. Food systems research has long been a component of many existing research fields, including nutrition, agriculture, environment, and global health; however, it is currently in a new state of development globally. Within the past decade, food systems research has evolved from being viewed simply as a component or an approach, to now being widely recognized as a distinct field. However, there has been limited public acceptance and funding for food systems research, resulting in limited work within this field and a lack of utilization of scientific evidence to create needed action (i.e. policy, programs, etc.) [5, 6].

Like the IDRC, research funders can contribute to building a field because of the unique role they play in the initiation, encouragement, support, and conduct of research. Through the use of specific research eligibility criteria, funders can directly influence the shape, direction, and legitimacy of research in a field [1, 3].

In this commentary, we provide an external evaluator's perspective on how well the Food, Environment, and Health (FEH) program has contributed to building the field of food systems research. We discuss the IDRC’s role in building the field of food systems research, based on a secondary analysis of findings from the recent FEH program evaluation [13]. For the secondary analysis, we used an existing field-building framework to highlight the strengths and challenges of the field-building approach used by the IDRC’s FEH program.

## Background

The IDRC is an organization that encourages, funds, and supports research in low- and middle-income countries (LMICs) to find meaningful and practical solutions to development problems [8]. One of the IDRC’s many research programs is the FEH program, made up of three subthemes: food systems, infectious diseases, and tobacco control. Within the food systems field, the FEH program’s focus is predominantly on research on the demand side of consumer environment-related policy interventions that promote or enable healthy and sustainable shifts in consumption to prevent chronic disease risk factors and improve population health. This has included developing, testing, and assessing public policies (e.g. fiscal policies, regulations on ingredients, marketing, labelling) that increase the consumption of fresh and minimally processed foods or decrease consumption of highly processed foods.

In 2019, a team at the University of Toronto led an external evaluation of the five-year FEH program using a participatory evaluation approach. Data collection occurred from May to July 2019, which coincided with the end of the FEH program. The summative evaluation’s goal was to provide evidence and recommendations for how the IDRC could improve the FEH program’s effectiveness to enhance its outcomes. The evaluation focused on six questions; however, this commentary will only reflect on findings related to the following evaluation question, which pertains to field-building:

- Given the context, risks and opportunities that emerged over the program period, how well has the FEH program implemented a strategic body of research programming in the thematic area of food systems research?

The evaluation applied qualitative and quantitative methods and triangulation of diverse data sources, including document reviews, 33 key informant interviews, a landscape assessment, and case studies. Key informants were recruited using purposive sampling drawing from a longer list provided by IDRC of 75 individuals inclusive of several stakeholder groups. The evaluation team achieved a representative response rate across all interviewee categories: 100% (11/11) response rate for IDRC staff; 50% (4/8) for donors and partners; 32% (12/37) for grantees; and 31% (6/19) for advisors/consultants/knowledge users. The participants had all received some sort of funding from IDRC or worked in partnership with them. Investigator triangulation was applied whereby two researchers were involved in conducting and reviewing the key informant interview data [2, 13]. The landscape analysis was used to frame and contextualize the results. The four case studies combined evidence drawn from key informant interviews and the document review [13]. The document review included a random selection of all project data sources dating from 1 April 2015 to 31 March 2019, with inputs from IDRC to ensure a representative sample. The final evaluation report and the interview data that this commentary draws upon were completed in September 2019. Data from the interviews and the case studies were included in the present analysis, and all quotes cited are illustrative of key informant responses.

## Discussion

The field-building framework outlined in Di Ruggiero et al. [4] provided an evidence-based approach through which to demonstrate how the IDRC has helped (or not) to build the field of food systems research. This framework was selected based on its relevance and applicability to field-building for a research funding organization based on prior empirical research [4]. The framework's underlying features (see Table 1) were first reviewed to gain a clear understanding of what the framework attempted to achieve. The evaluation results were then analysed deductively, with reference to pre-identified features from the framework. The field-building framework posits that several key features need to be present for a new field of research to be built successfully and to have the potential to influence policy, program, and practice decision-making.

**Table 1:** Key features from the field-building framework outlined in Di Ruggiero et al. [4]

Key feature	Definition
Support and awareness of the field	Creating enhanced awareness, profile, funding, relationships, and connectivity for the field
Organized funding	An ongoing or sustained flow of resources to maintain new ideas and investigations
Team-level capacity-building	The process by which teams of individuals develop, strengthen, and retain skills, knowledge, tools, and other resources needed to conduct and sustain the research
Multilevel activity	Activities occur at various levels (individual, organizational, and systemic), with horizontal and vertical coherence in action to support the funding, conduct, and use of research in a given area
Influential scientific leadership	A knowledge base of credible, policy-oriented, and action-oriented evidence by a key group of researchers
Setting standards and exemplars	Being viewed as a key source of external legitimacy, which others look to for examples and standards on how to proceed in similar situations
Supportive environments	The role of physical, social, and political environments in supporting the research being conducted and its uses

### The IDRC’s current approach to field-building through FEH

#### Support and awareness of the field

Creating enhanced awareness, support, and connectivity is crucial when building a field of applied research [4]. As summarized in our evaluation of the FEH program described below, the IDRC has been recognized as one of the leaders in building the field of food systems research [13]. As such, they have brought legitimacy, attention, and influence to research agendas within the field. Although this was not explicitly mentioned as a goal of the FEH program, several key FEH approaches have contributed to creating support and awareness for food systems research.

The IDRC’s field-building efforts have garnered the attention of grantees and other funding bodies—as illustrated by a grantee’s quote, “IDRC is about the only body that is interested in food systems at that time, and even now. I get the impression that they are somehow a little bit the forerunners.” Most notably, the FEH program is funding globally relevant food systems research on sugar-sweetened beverage (SSB) tax legislation to build an emerging knowledge base on the effectiveness of these policies for non-communicable disease (NCD) prevention in LMICs. In South Africa, the FEH program contributed to the implementation of a SSB tax by supporting a research team that had strong policy advocacy connections and provided context-specific evidence. The FEH program has had a significant impact “not just in the field but to move that field forward and get it on agendas of folks who are resourced enough to do something about it […] in this respect, I think they’ve done brilliantly.”

The FEH program also acted on evaluation recommendations and learnings from past IDRC programs, including the Non-Communicable Disease Prevention program (NCDP) and Ecosystems and Human Health (EcoHealth), which each had strategic objectives to build the fields of research for their respective thematic areas. One of the most influential recommendations that the IDRC employed with the FEH program is developing networks and South-to-South collaborations, which contributed to their systems approach. South-to-South collaboration refers to the exchange of resources, technology, and knowledge between countries in the Global South [14].

Similarly, the FEH program is recognized as improving awareness and employing a prevention orientation to the food systems field. A FEH staff conveys this by stating, “We can’t claim credit for where the ‘field’ is now, but there definitely has been a change in the discourse when they’re talking about the global epidemic of NCDs, it’s shifted […] it is much more recognized, but there’s been a shifting in the framing of the problem away from individual behaviours […]. I think when a lot of our research partners, particularly some who are very vocal or very influential in those high-level policy spaces are helping to push out that message, you know, I do think we are part of that change, that building of that field.”

### Organized funding

The framework presented by Di Ruggiero et al. [4] states that an ongoing or sustained flow of resources is important to maintain new ideas and investigations when building a new field of research. The approach taken by the FEH program is unique in that not all networks and project locations are at the same level of research capacity academically or within government and civil organizations. In response to this, the FEH program has taken on various funding modalities (e.g. co-funding, parallel funding), differing in terms of structure and flexibility, to tailor their approach to different geographical and institutional contexts.

The program’s approach to partnering involves offering flexible funding modalities that flow funding to LMICs and emphasize sustainability. In doing so, the IDRC, through the FEH program, has been viewed as a highly valued partner by other research donors. The donors interviewed during the evaluation felt that their interests in strengthening the capacities in LMICs were reflected in the funding options for the program. This is illustrated through one donor/partner’s perspective: “[There were] common principles with IDRC in building research capacity in LMIC and […] ensuring that the LMIC research teams have a leadership role including management of finances, […] something that we’re just not able to do directly given [our] mandate […] so we highly value that piece of our relationship with IDRC.”

The IDRC’s FEH program has invested considerably in food systems research, which has contributed to the field's progress. By funding research in this field, the IDRC has taken a stance that this research area is both needed and meaningful, signalling to other research for development funders the importance of increased focus.

### Influential scientific leadership by a core cadre

To successfully build a field of research, there needs to be a strong knowledge base of credible, policy-oriented, and action-oriented evidence by a key group of researchers [4]. The IDRC’s success in building fields of research in the past, such as with the EcoHealth program, gives them significant credibility acquired through experience [11]. This credibility and legitimacy have been recognized by grantees and other research funders, including in the field of food systems research. The FEH program is particularly known for funding projects that are globally relevant and driven by goals of policy and practice change. The evaluation found that this has created a niche of sorts where the FEH program is recognized for funding action-oriented research at the intersections of food, health, and the environment. The IDRC is one of the only funders supporting NCD and food systems research, making them a thought leader in the emerging field of food systems research. Describing the scientific excellence that the program has, one FEH staff highlighted that “one of the greatest achievements for me in this programming cycle is the food systems portfolio, the richness, the diversity, its global coverage […] with very short years we were able to develop some great projects and it’s producing some emerging results.”

### Team-level capacity-building

Supporting the building of new research fields requires that teams of individuals develop, strengthen, and retain skills, knowledge, tools, and other resources needed to conduct and sustain the research [4]. We found that the FEH program is contributing to capacity-building within and between countries. Moreover, IDRC’s FEH program is building a strong foundation for southern leadership and communities of practice.

The FEH program provides support to LMIC researchers, and many individuals stated that they feel valued because they are viewed as equals through interactions with the IDRC, such as during network collaboration events and capacity-building training. For example, the FEH program facilitated a joint proposal development workshop collaboratively with scientists from 12 countries and established a community of practice on food systems research.

Concerning the FEH program’s development of capacity-building and collaboration, one FEH staff highlighted that “for capacity-building [IDRC brought] together researchers and policy actors within and between countries in a region and beyond for learning and capacity-strengthening.” This sentiment was reflected by many other grantees, one stating, “FEH’s ability to create coalitions or networks of researchers who we could link with our domestic researchers is valuable just from a pure kind of information sharing or increasing new knowledge and increasing capacity around the world on food systems or food policy issues.” Not only has the FEH program made intentional efforts to create networks, training, and capacity-building in the regions where research is being conducted, but they have also tried to contribute to sustaining that capacity. During the evaluation interviews, one FEH advisor said, “There’s enough there that if everything was to disappear in the near future in terms of IDRC investments, there’s some actual sustained capacity for continued effort.”

### Multilevel activity

To build a field of research, activities need to occur at various levels (individual, organizational, and systemic), with horizontal and vertical coherence in action to support the funding, conduct, and use of research [4]. Based on feedback from prior internal program evaluations, the FEH program developed regional strategies specifically tailored to align with the research field’s stage of development within the given region. For example, the field of food systems research is becoming established in Latin America, the Caribbean, and South Africa, with evidence of strong capacities and direct FEH contributions to building interregional collaborations in research and policy. However, in Africa, Asia, and the Middle East, food systems research is not as well developed. In response to this, the FEH program aimed to strengthen multi-country, multi-institutional South-South collaborative research with leadership and support from a few strong institutions in South Africa, Kenya, and Lebanon. In part, this is because the IDRC took an intentional approach tailored to the needs of each region. A tailored approach was adopted because each region had differing needs. In regions with less capacity, it was an issue of limited critical mass and research outputs, and a less favourable policy, as further described in the full evaluation report [13].

### Standards and exemplars

Concerted action to develop a field of research needs to involve setting standards and exemplars that have external legitimacy so that others can look to these when determining how to proceed in similar situations [4]. The FEH program has contributed to the setting of standards through their involvement with INFORMAS (International Network for Food and Obesity/noncommunicable diseases Research, Monitoring and Action Support) [7]. INFORMAS is a global network of organizations and researchers with the goal of monitoring, benchmarking, and supporting actions to improve and increase healthy food environments and subsequently reduce the rates and burdens of NCDs [7]. The IDRC has funded and supported projects that were implementing various INFORMAS approaches. One FEH partner said, “I co-funded, along with FEH, the Brazil INFORMAS and we’re using that data enormously to push, create policies, the same in some number of countries […] in Chile [FEH] is funding more evaluations and the same in Mexico. So [FEH] has helped because we’ve used these evaluations to influence other countries.”

As previously mentioned, the FEH program has set significant exemplars and gained external legitimacy through its investment in impactful SSB taxation policy research. The FEH program's efforts in supporting researchers to interact with policy-makers, generate evidence, and disseminate research will likely serve as an exemplar to other research funders looking to push SSB taxation policy agendas.

However, in terms of setting an exemplar for defining the field of food systems research, the diverse thematic focus beyond food systems within the FEH program may have impacted its ability to represent a strategic body dedicated specifically to food systems research. The FEH program originated from the merging of two previous IDRC programs, NCDP and EcoHealth. Although this merger brought forth several benefits, it has also resulted in the FEH program being broadly spread in thematic areas (food systems research, infectious diseases, and tobacco control). The dispersion of effort across issues beyond food systems may have reduced the FEH’s potential to be an even stronger exemplar for food systems research programming.

### Supportive environments

Di Ruggiero et al. [4] state that to build a field of research, supportive physical, social, and political environments are needed to recognize and value this research in peer-reviewed granting systems, publication systems, program efforts, and policy agendas. The field of food systems research is not receiving sufficient attention and support within most governments or from the broader policy landscape [5]. Since the role of food systems research in influencing policy is not always understood or on political agendas, the evaluation found that it was difficult for the FEH program to attract funding partners. As illustrated by one FEH staff, “Part of the external reason for lack of partnership on the NCD prevention […] or the food systems front is we’re not receiving the attention they deserve […] it’s not understood—the role of research in advancing policy and these issues continue to be, or particularly up until now, not regarded as issues for LMICs.”

The political environment within some regions was not entirely conducive to applying research findings. For example, one FEH-funded project focused on measuring and benchmarking food environments and policies in Latin America (Mexico, Chile, and Guatemala) found a different level of uptake in Mexico compared to the other two countries. In Mexico, the obesity prevention policies have faced some opposition from the food industry and policy-makers, which initially impacted the uptake and utilization of the research evidence. However, since the completion of the evaluation, a new law was published in Mexico that supported this research's major guidelines and recommendations, demonstrating that the political environment for food systems research may indeed be shifting. In contrast to what the field-building framework presents, it can be argued that unsupportive political environments can actually be an essential driver for engaging in food systems research and building the field to change political and public acceptance or perceptions. For food systems to be viewed as an important issue for policy and program agendas within LMICs, a critical mass of research within the field is needed.

## Conclusion

Addressing the challenges faced by populations in LMICs, such as NCDs and consumer food environments, requires joint effort at many levels. Recognizing the need to respond to barriers facing these populations in food systems research, the IDRC’s FEH program has provided needed funding, legitimacy, and guidance for this field. The FEH program has successfully laid the groundwork for the field of food systems, in particular research related to consumer food environments, and the program efforts and components align with six of the seven features deemed necessary to build a field as per the framework outlined in Di Ruggiero et al. [4]. The FEH program has improved support for and awareness of food systems research, provided organized funding, supported team-level capacity-building, and introduced multilevel activities, and has strong scientific leadership and has set standards and exemplars. We found that the external environment for food systems research was not entirely conducive to conducting food systems research during the first four years of the FEH program. Yet, despite this, the program has still been successful in contributing to building the field of research. This suggests that the feature of supportive environments may not always be necessary to build a field of research initially; consequently, the presence of all the required field-building components deemed necessary may not be needed at the outset, as was originally thought. However, supportive environments may be more critical in ensuring the field’s sustainability, creating a critical mass of capacity and building communities of practice. Although new knowledge about the importance of food systems has been brought to light by COVID-19, this was not considered because the pandemic had not yet emerged when the evaluation of the FEH program was conducted. Nevertheless, the FEH program approach to building the field of food systems research may serve as an exemplar for other funding agencies looking to develop strategic research programming.

## Data Availability

The data generated and/or analysed during the current study are not publicly available due to ethical considerations but may be available from the corresponding author on reasonable request.

## Abbreviations

EcoHealth: Ecosystems and Human Health
FEH: Food, Environment, and Health program
IDRC: International Development Research Centre
INFORMAS: International Network for Food and Obesity/noncommunicable diseases Research, Monitoring and Action Support
LMIC: Low- and Middle-Income Countries
NCD: Noncommunicable diseases
NCDP: NonCommunicable Disease Prevention program
SSB: Sugar-Sweetened Beverage
